# Supplementary material for: Overexpression of Both Human Sodium Iodide Symporter (NIS) and BRG1-Bromodomain Synergistically Enhances Radioiodine Sensitivity by Stabilizing p53 through NPM1 Expression
Source: Int J Mol Sci. 2023 Feb 1;24(3):2761. doi: 10.3390/ijms24032761 (PMC9917390; doi:10.3390/ijms24032761)
Supplement: Supplementary file 1 [file ijms-24-02761-s001.zip › ijms-2157918-supplementary.pdf]

Supplementary Figure S1

A

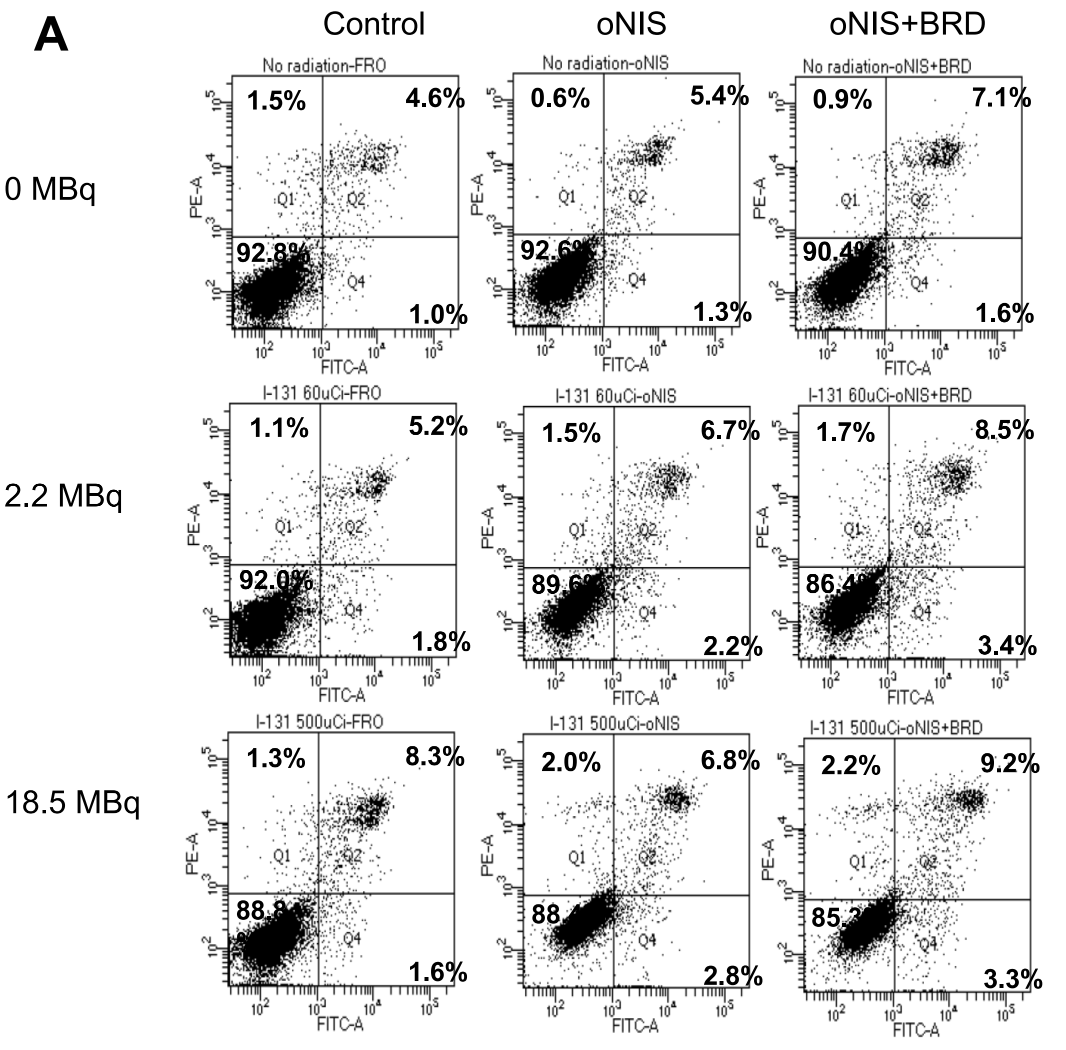

B

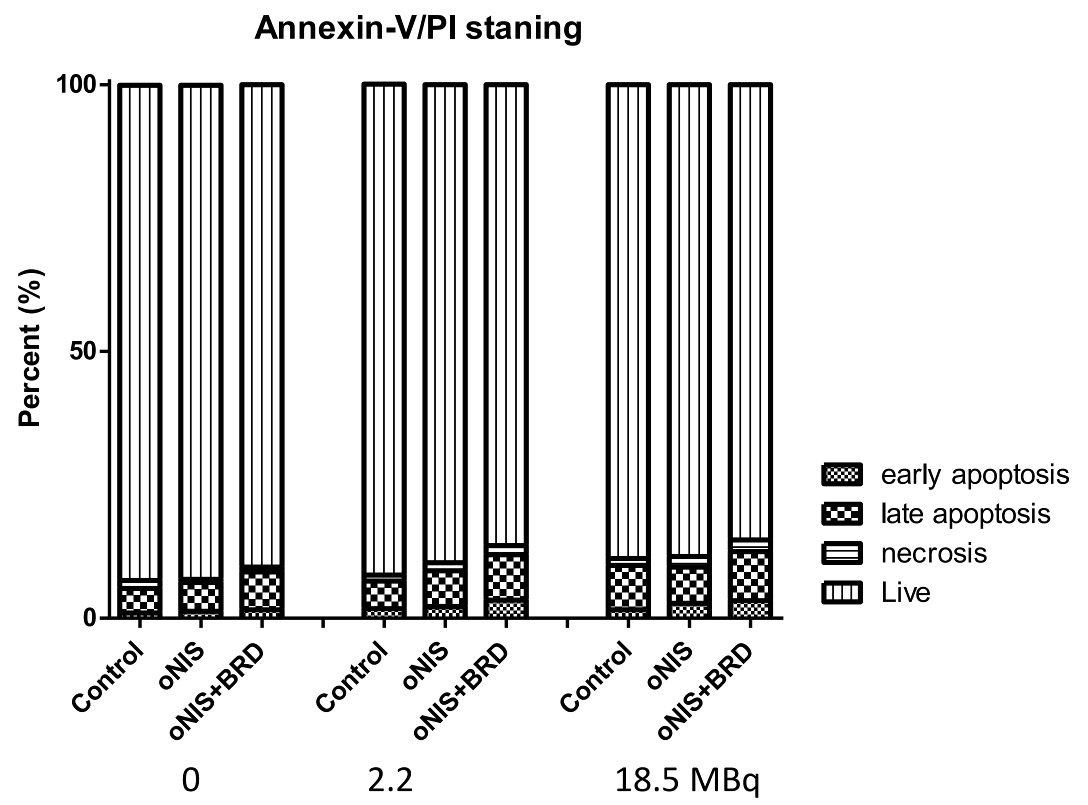

C

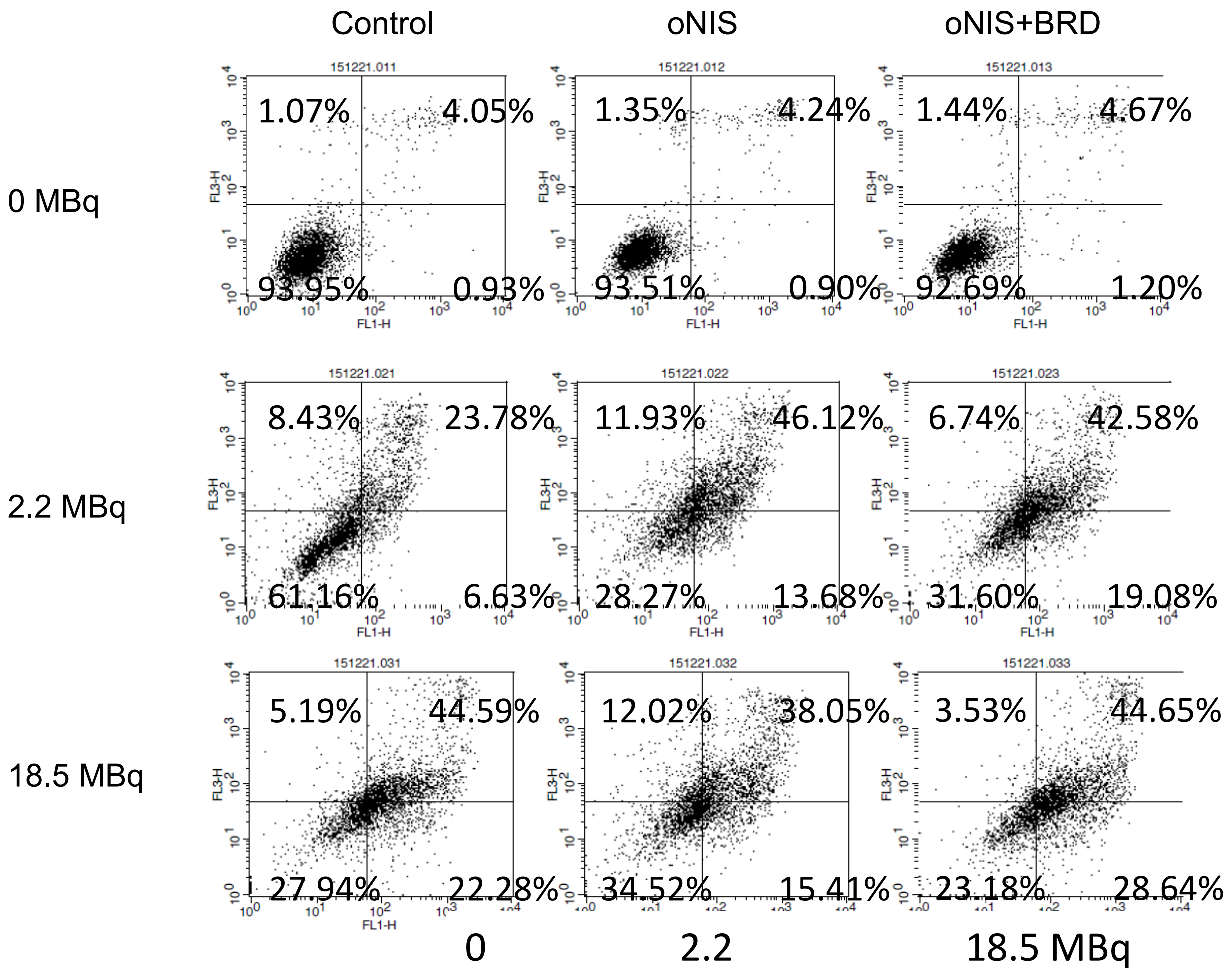

D

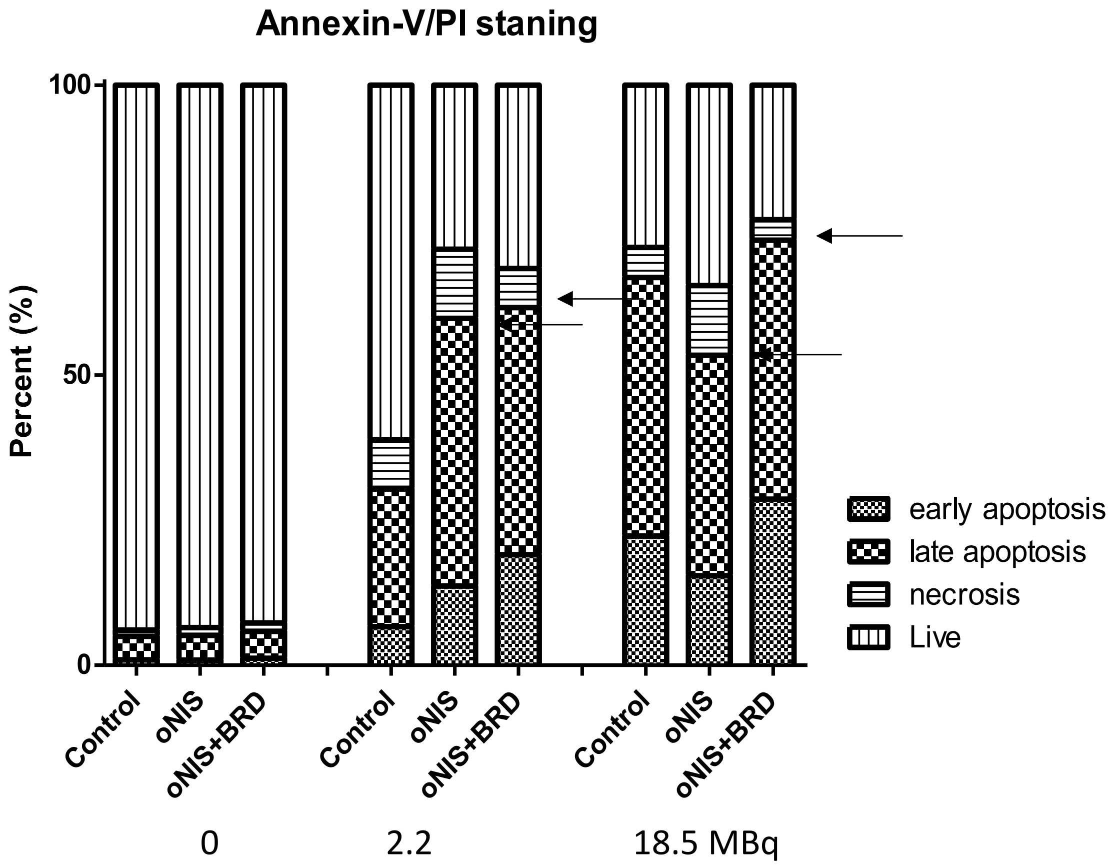

Figure S1. Apoptosis level after I-131 treatment in control, oNIS, or oNIS+BRD cells. (A) Representative images of Annexin-V/PI results (7 hr incubation with I-131, 24 hr extra incubation after wash-out). (B) Analysed bar graph from (A). (C) Representative images of Annexin-V/PI results (5 days incubation without wash-out). (D) Analysed bar graph from (C).

Supplementary Figure S2

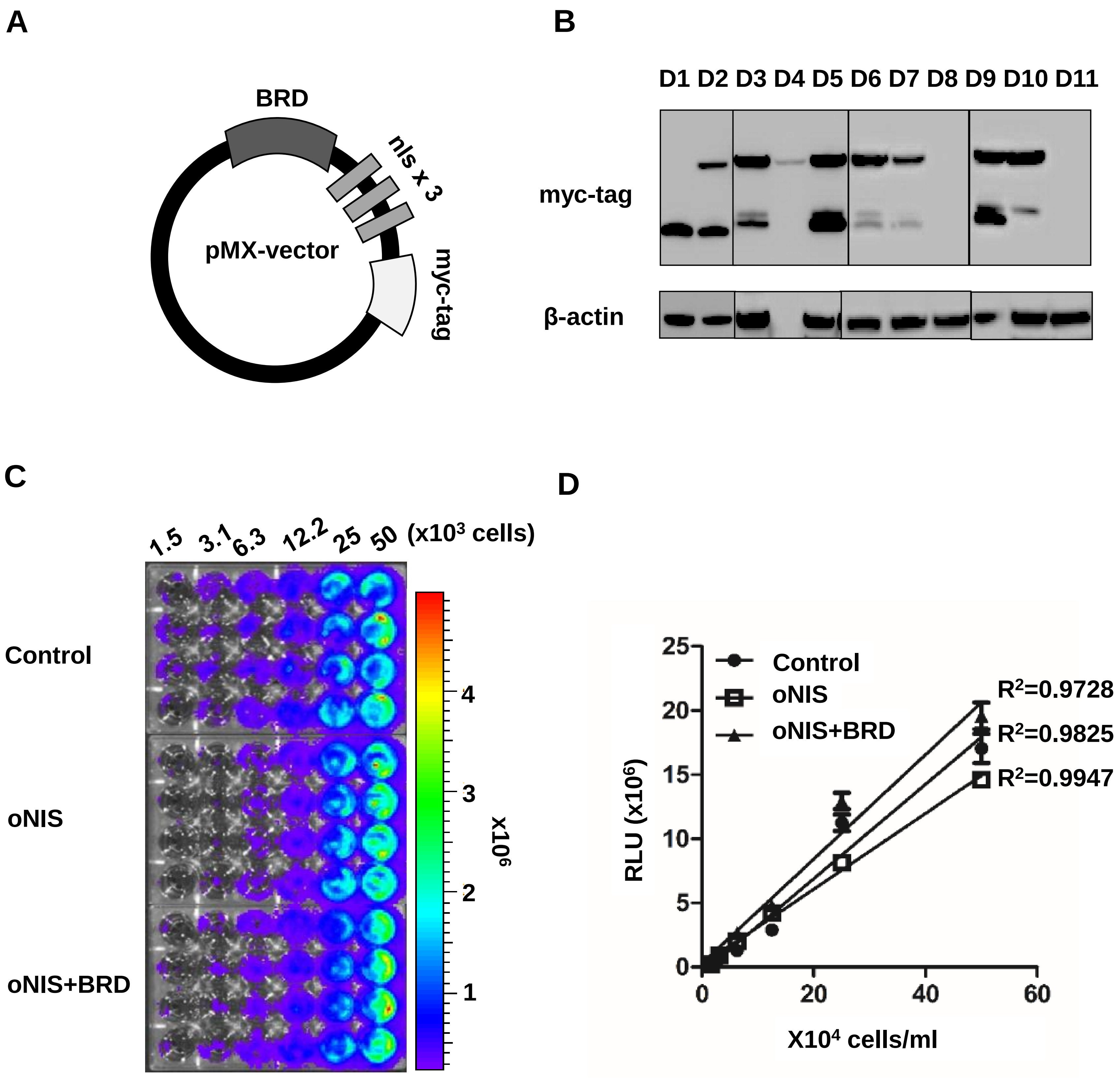

Figure S2. Characterization of control, oNIS, and oNIS+BRD cell lines. (A) Map of BRG1-BRD overexpressing vector with myc-tag. (B) Expression of myc-Tag was evaluated by Western Blot. Among these cell clones, the D10 clone was designated as oNIS+BRD cells through monoclonal selection. (C) The bioluminescent signal from IVIS 100 correlated with cell number. The scale bar represents the peak signal in photons/s/cm2/sr. (D) Luciferase activity of cells was measured by luciferase assay.

Supplementary Figure S3

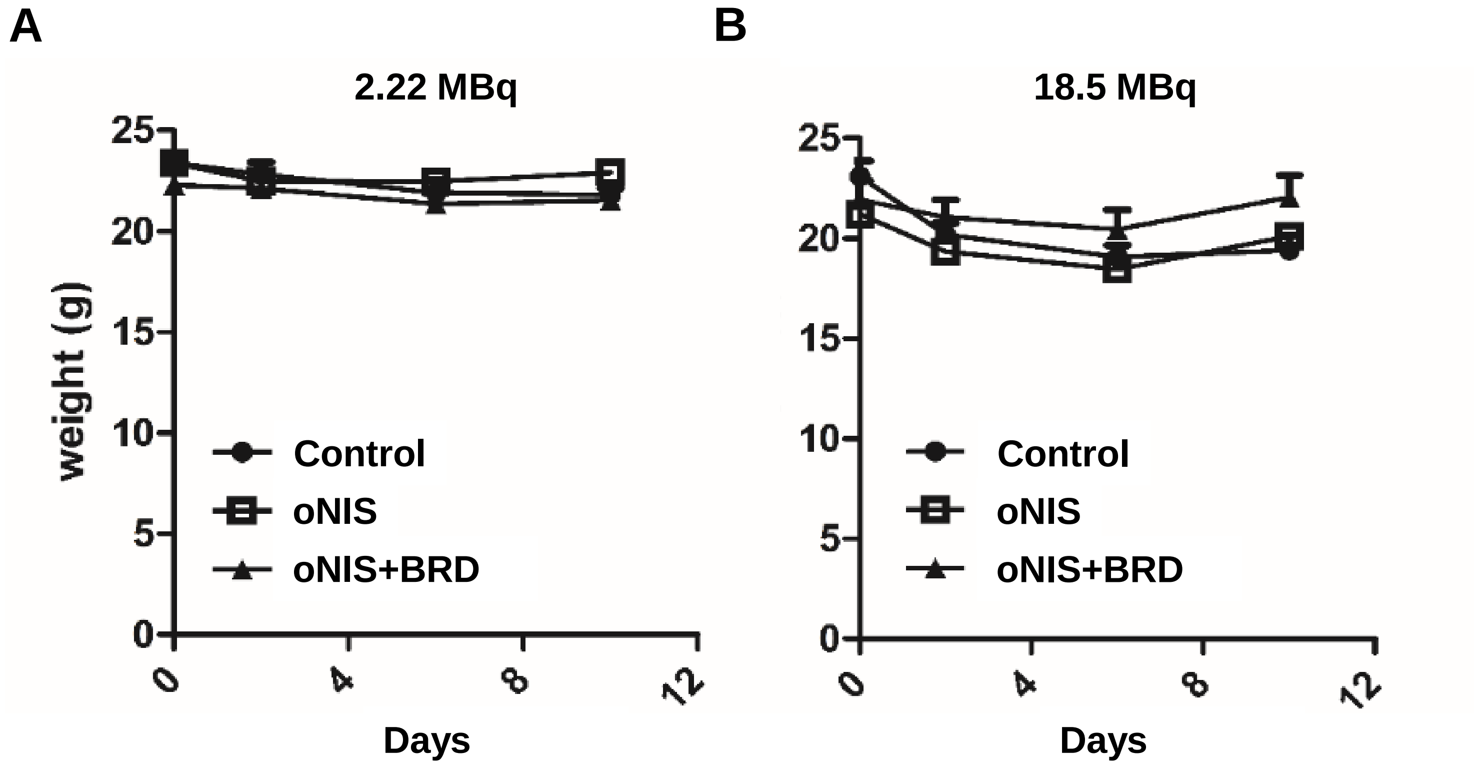

Figure S3. Changes in mouse body weight after I-131 treatment. (A) Body weight was measured at the same time point for the 2.22 MBq of the I-131 treatment group. (B) Body weight was measured at the same time point for the 18.5 MBq of the I-131 treatment group.

Supplementary Figure S4

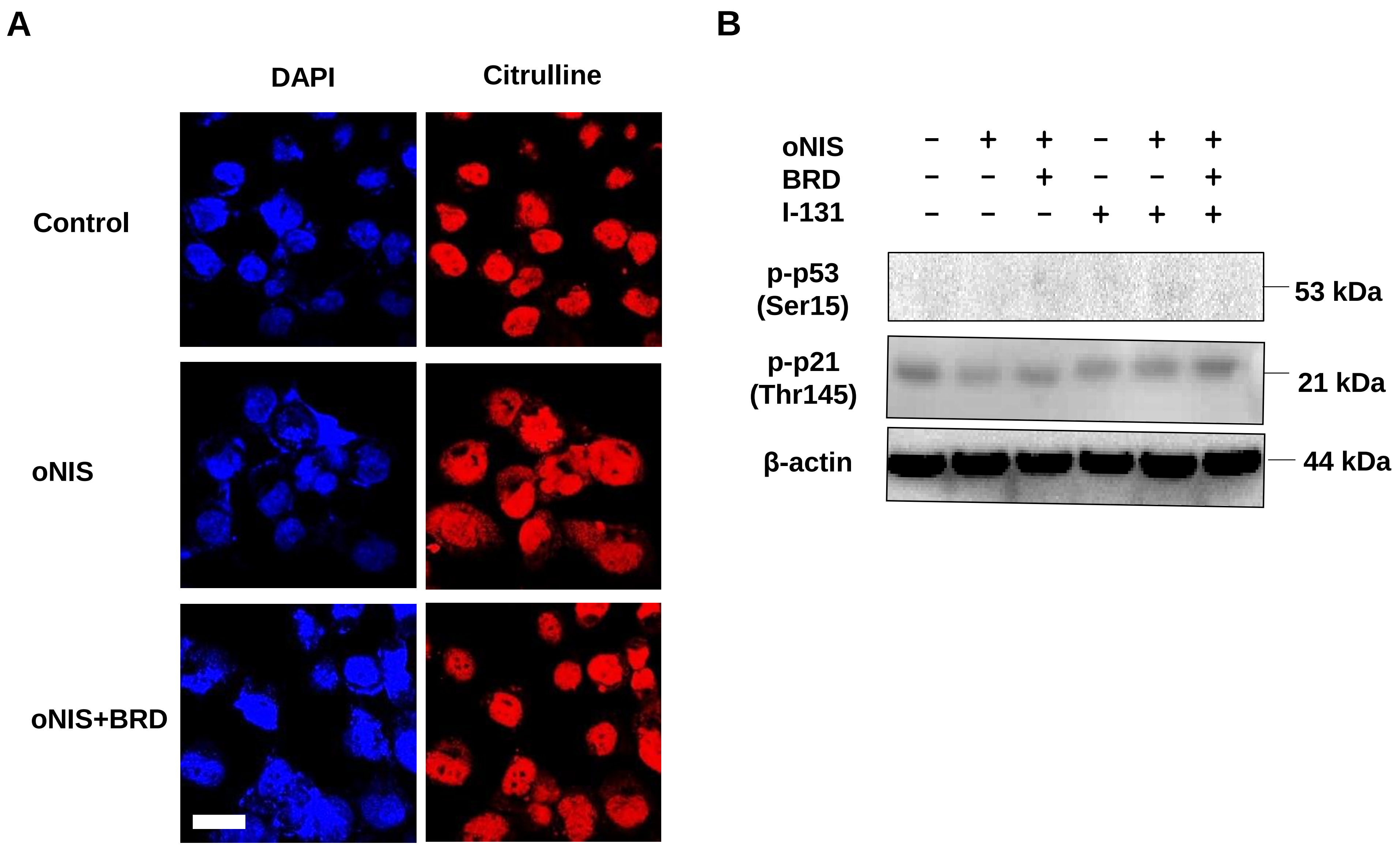

Figure S4. Measurement of histone citrullination after low-dose I-131 treatment and phosphorylated p53 and p21 after high dose I-131 treatment. (A) Immunocytochemistry of histone citrullination after low-dose (2.22 MBq) I-131 treatment. (B) Measurement of apoptosis-related proteins after high-dose (18.5 MBq) I-131 treatment. Western Blot analysis of phosphorylated tumor suppressor proteins. Scale bar=30 μm.

Supplementary Figure S5

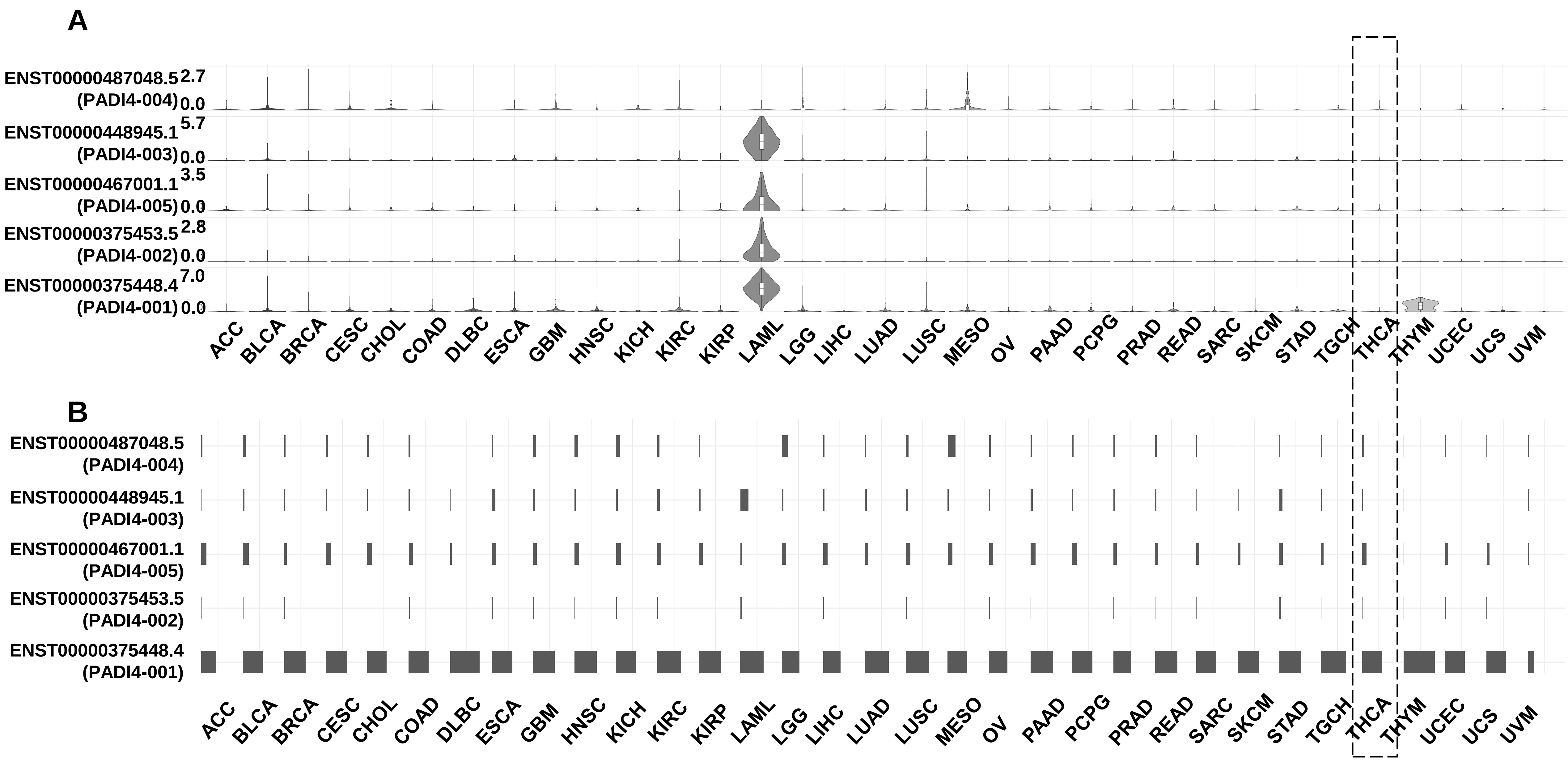

Figure S5. The profile of the PAD4 expression distribution, and the isoform usage in multiple cancer types. (A) The expression distribution of PAD4. PAD4 isoform 1 and 4 have basal expression in most of cancer types. LAML and THYM have relatively strong expression of PAD4 isoform 1. (B) The isoform usage of PAD4. PAD4 isoform 1 is the major isoform followed by isoforms 5 and 4. There is relatively less expression of isoform 1 in THCA than most of other cancer types. THCA is marked with dashed box.

Supplementary Figure S6

A

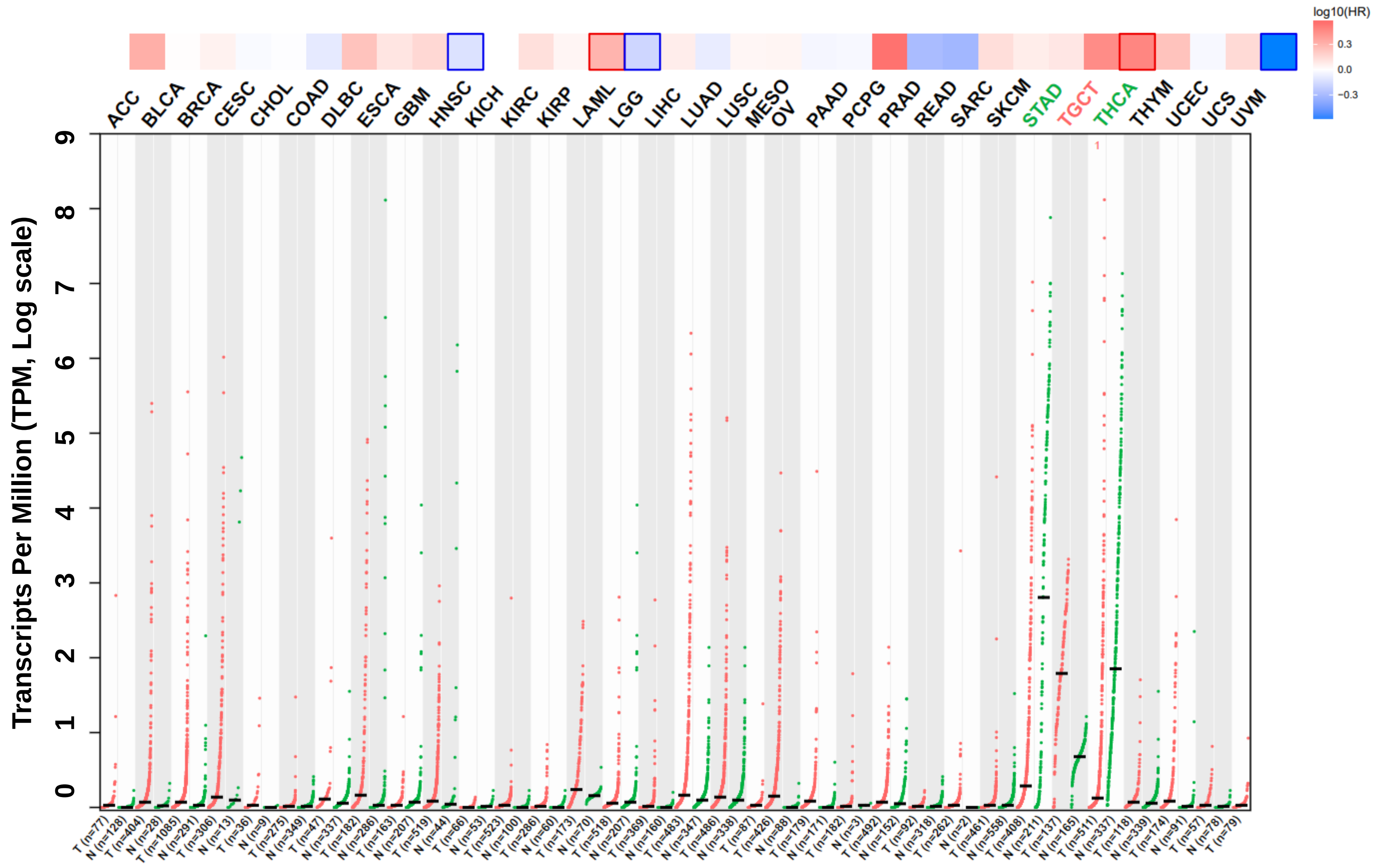

B

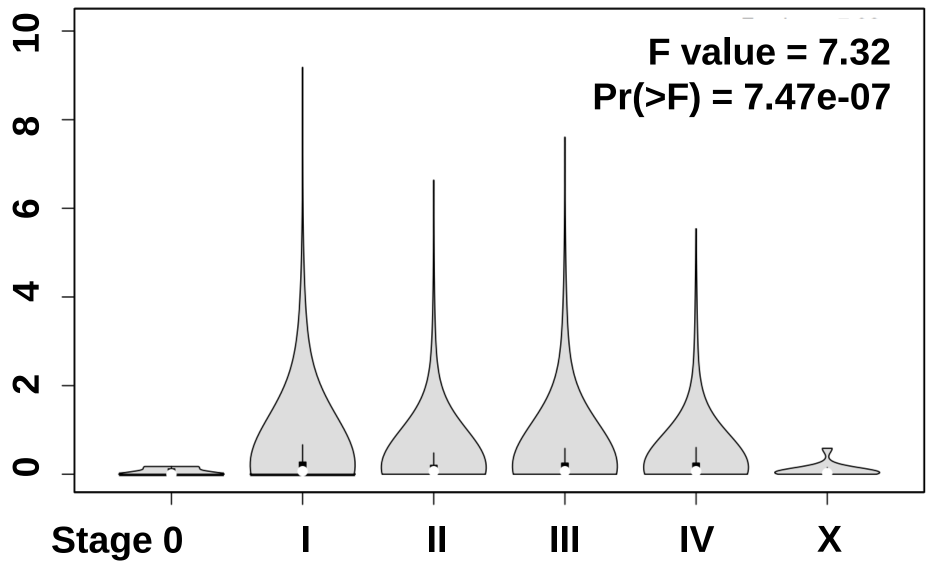

C

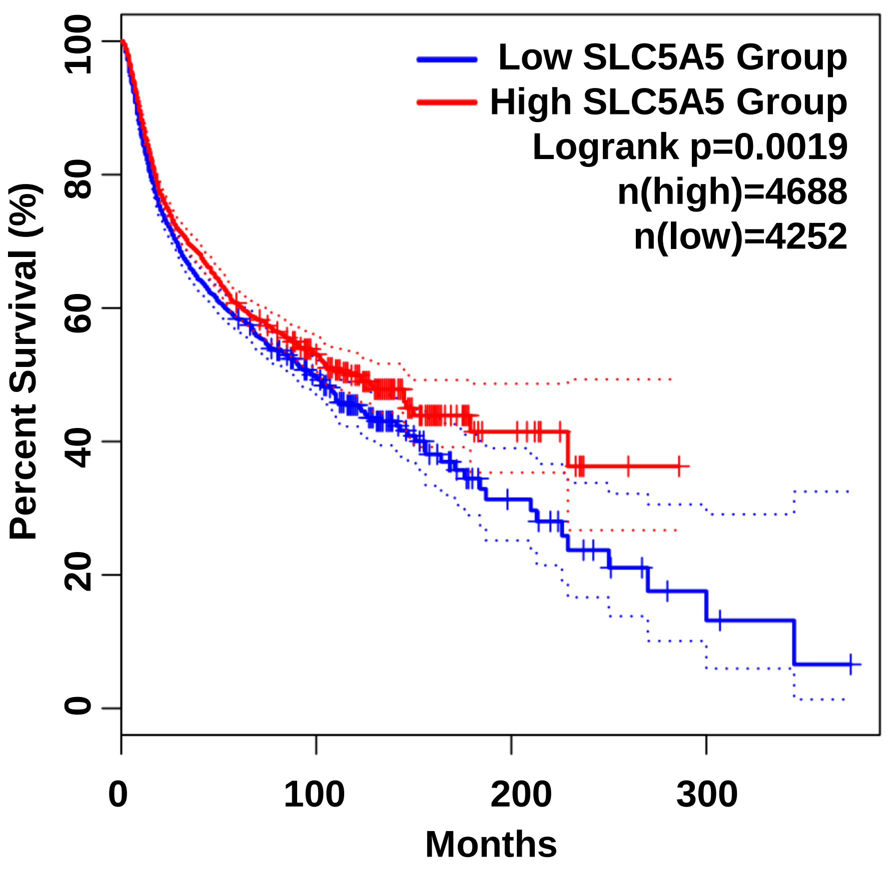

Supplementary Figure S6 (Continued)

Figure S6. Gene expression levels of SLC5A5, encoding NIS, in normal and tumor tissue and patients' survival rates depending on its expression. (A) TCGA NIS gene expression levels across different cancer types, comparing tumor expression levels (red dots) to normal tissue (green dots). Tumor abbreviations in red have a statistically significant increase in SLC5A5 gene expression compared to normal tissues, whereas abbreviations in green have higher levels of SLC5A5 gene expression in normal tissues. Abbreviations in black show no statistically significant effect. The survival map of gene SLC5A5 shows significant results in HNSC, LAML, LGG, and THCA (above the graph). (B) Gene expression levels of SLC5A5 in different stages of listed types of cancer. (C) Disease-free survival analysis of SLC5A5 gene expression from listed types of cancer; longrank test, *p=0.0019.
ACC; Adrenocortical carcinoma, BLCA; Bladder Urothelial Carcinoma, BRCA; Breast invasive carcinoma, CESC; Cervical squamous cell carcinoma and endocervical adenocarcinoma, CHOL; Cholangio carcinoma, COAD; Colon adenocarcinoma, DLBC; Lymphoid Neoplasm Diffuse Large B-cell Lymphoma, ESCA; Esophageal carcinoma, GBM; Glioblastoma multiform, HNSC; Head and Neck squamous cell carcinoma, KICH; Kidney Chromophobe; KIRC; Kidney renal clear cell carcinoma, KIRP; Kidney renal papillary cell carcinoma, LAML; Acute Myeloid Leukemia, LGG; Brain Lower Grade Glioma, LIHC; Liver hepatocellular carcinoma, LUAD; Lung adenocarcinoma, LUSC; Lung squamous cell carcinoma, MESO; Mesothelioma, OV; Ovarian serous cystadenocarcinoma, PAAD; Pancreatic adenocarcinoma, PCPG; Pheochromocytoma and Paraganglioma, PRAD; Prostate adenocarcinoma, READ; Rectum adenocarcinoma, SARC; Sarcoma, SKCM; Skin Cutaneous Melanoma, STAD; Stomach adenocarcinoma, TGCT; Testicular Germ Cell Tumors, THCA; Thyroid carcinoma, THYM; Thymoma, UCEC; Uterine Corpus Endometrial Carcinoma, UCS; Uterine Carcinosarcoma, UVM; Uveal Melanoma.

Supplementary Figure S7

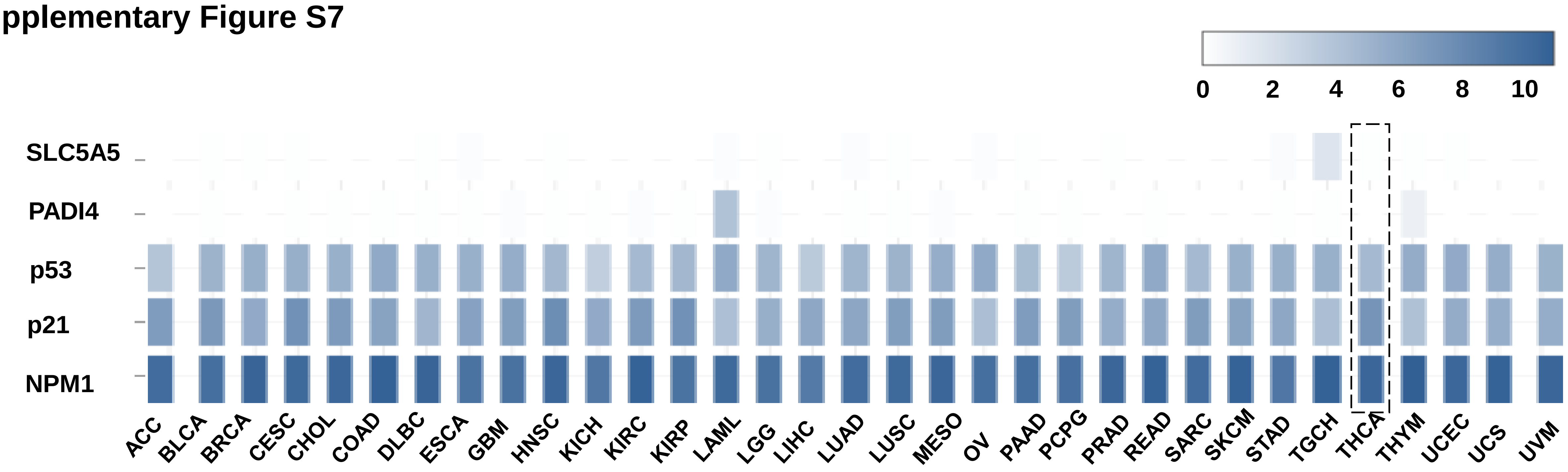

Figure S7. Multiple genes comparison in tumor TCGA data. PAD4 is strongly expressed in LAML and THYM cancer types. NPM1, p21, an p53 are strongly expressed in most of cancer types overall. THCA is marked with dashed box.
